# Keeping up the Pace: A Case of Complete Heart Block

**DOI:** 10.7759/cureus.37606

**Published:** 2023-04-15

**Authors:** Kevin Maraj, Varsha A Persad, Aaron Hinds

**Affiliations:** 1 Internal Medicine, Port-of-Spain General Hospital, Port-of-Spain, TTO; 2 Adult Medicine, Port-of-Spain General Hospital, Port-of-Spain, TTO

**Keywords:** trinidad, electrocardiogram, bradycardia, pacemaker, complete heart block

## Abstract

We present the case of a 56-year-old lady who presented with symptomatic bradycardia and was outsourced for permanent pacemaker implantation. The discussion that follows highlights the growing need for permanent pacemakers globally and in Trinidad and Tobago, as well as the stepwise approach needed for investigating patients with symptomatic bradycardia. Finally, recommendations are made for policy changes needed at the national level.

## Introduction

Atrioventricular blocks are the leading cause of pacemaker implantation globally, with an increased rate of implantation being attributed to an increase in global life expectancy and associated degeneration of cardiac conduction pathways [1]. These findings were supported by Mond and Proclemer who noted that implantation rates have increased in almost all countries [2] and the European Society of Cardiology (ESC), which noted that approximately 1 million devices are implanted on a global scale annually [3]. The ESC further advocates a stepwise approach to investigating bradycardia utilizing a thorough history and examination supplemented with the electrocardiogram and cardiac imaging [3]. This case study aims to highlight this stepwise approach and the discussion that follows will highlight the need for augmented pacemaker services in developing countries.

## Case presentation

**Figure 1:**
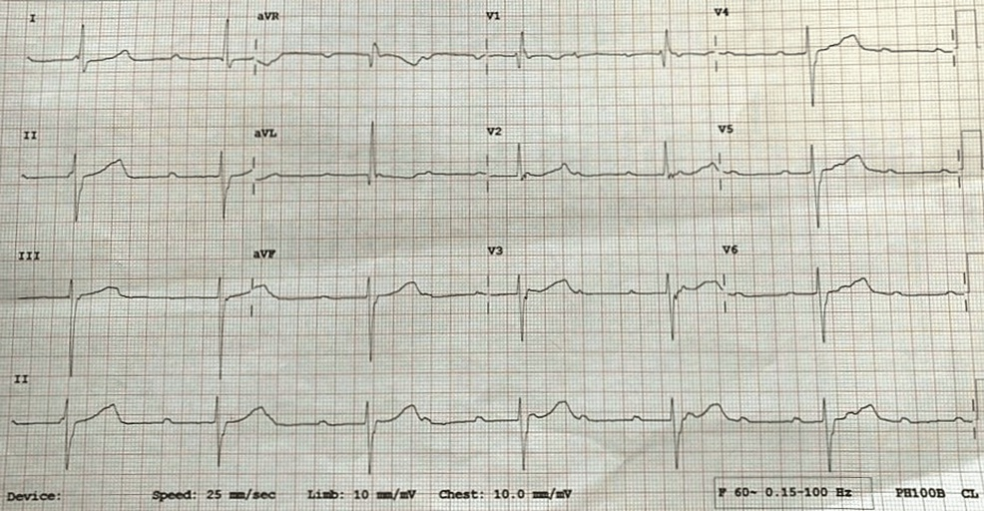
ECG showing a complete heart block

We present the case of a 56-year-old lady who presented with a one-day history of generalized weakness and dyspnea on a background of type 2 diabetes mellitus, hypertension, previous ischemic stroke, and previous coronavirus disease 2019 (COVID-19) infection. Her drug history included Nifedipine Sustained Release 40 mg po od, gliclazide 80 mg po od, clopidogrel 75 mg po od, and rosuvastatin 20 mg po od. There was no history of oral or ophthalmic beta-blocker use and no history of hypothyroidism. Family history was positive only for type 2 diabetes mellitus. Physical examination revealed a blood pressure of 194/82 mmHg, pulse rate of 34 beats per minute, respiratory rate of 16 breaths per minute, temperature of 36.2 degrees Celsius, oxygen saturation of 97% on ambient air, and random capillary blood glucose of 195 mg/dl. Her apical rate was regular at 36 beats per minute with no added heart sounds or murmurs. Orthostatic blood pressure and pulse measurements were not done. The 12-lead electrocardiogram (ECG) showed a complete heart block with a ventricular rate of 37 beats per minute (Figure 1).

Serial ECGs during the course of her admission consistently showed a complete heart block while transthoracic echocardiography revealed mild concentric left ventricular hypertrophy, grade 1 diastolic dysfunction, and a left ventricular ejection fraction of 86.3%. Blood investigations, including thyroid function tests and electrolytes, were non-contributory.

**Figure 2:**
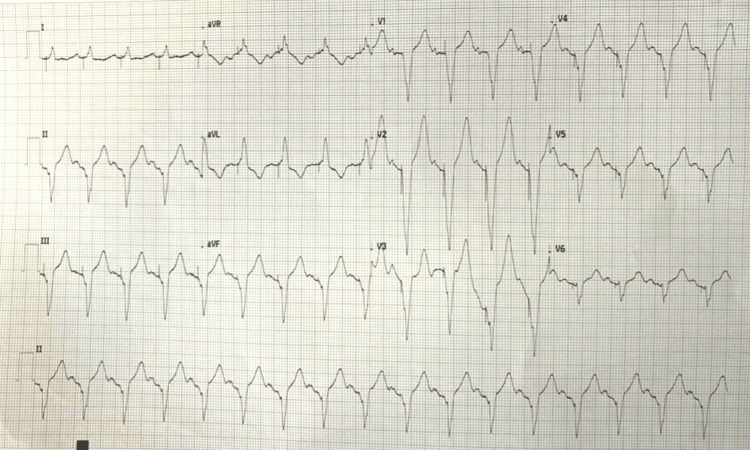
Postoperative ECG with pacing spikes

She was reviewed by the cardiology service, as her dyspnea persisted, and was subsequently outsourced to the regional cardiology hospital for permanent pacemaker placement. This was done as a day case and the patient was discharged from the hospital on the following day with a manual pulse of 98 beats per minute and resolution of her dyspnea. Her post-procedure ECG showed pacing spikes and a ventricular rate of 100 beats per minute as shown in Figure 2.

## Discussion

A worldwide survey involving 61 countries in 2009 found that over one million pacemakers were implanted globally with virtually all countries showing an increase in the number of implants over the last four years [2]. The rate of permanent pacemaker implantation in Trinidad and Tobago rose from 39 to 103 per million population between 2005 and 2009 with the main indications being high-degree atrioventricular blocks and sick sinus syndrome [4]. This implantation rate remains well below the international standard with the rate of implantation in the United Kingdom in 2008 being 511 per million [4]. The increasing rate of permanent pacemaker implantation in Trinidad and Tobago is likely to be a result of improving socioeconomic conditions and an aging population as suggested by Mkoko et al. in their analysis of cardiac arrhythmias in low and middle-income countries [5]. This disparity in implantation rates suggests that there is a need, at the national level to augment pacemaker implantation services. This includes bolstering the number of cardiac electrophysiologists, the number of centers offering cardiac electrophysiology services, and the availability of pacemakers.

**Figure 3:**
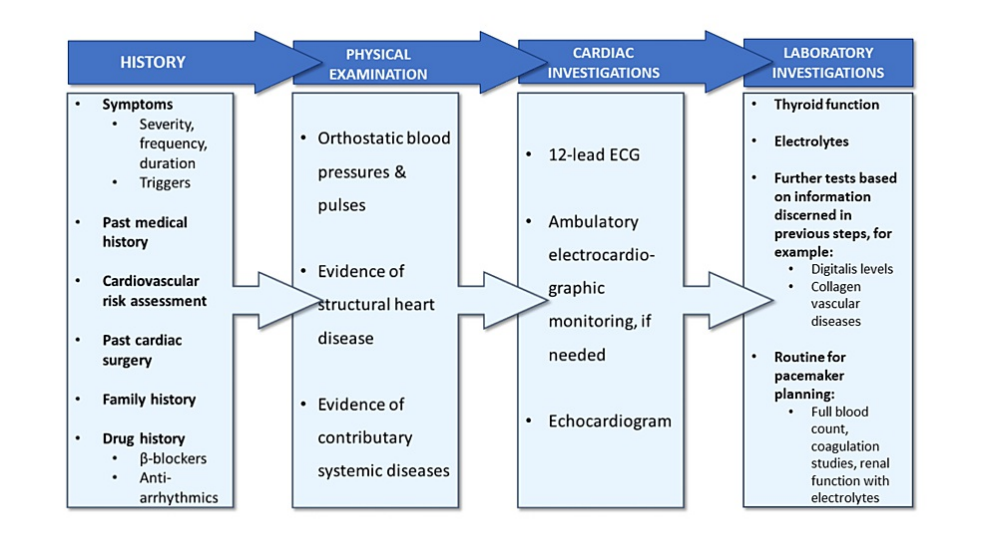
Outline of a stepwise approach to investigate patients with bradycardia

The ESC recommends a stepwise approach when investigating patients with documented or suspected bradycardia, which begins with a thorough history and examination [3]. The history should include a family history as well as a cardiovascular risk assessment, past medical history, and the frequency, severity, and duration of symptoms while the drug history should explore possible culprits, including beta blockers and antiarrhythmics [3]. The drug history must be particularly thorough with respect to ophthalmic beta blockers, which are known to cause bradycardia such as timolol [6,7]. The physical examination should include a search for underlying structural heart disease and systemic disorders as well as orthostatic changes in pulse and blood pressure while investigations should include an ECG, ambulatory electrocardiographic monitoring if bradycardia is intermittent and imaging to evaluate for potential structural heart disease [3]. Laboratory investigations should include thyroid function tests as well as tailored investigations if secondary causes are suspected [3]. This stepwise approach will also allow for reversible causes of bradycardia such as adverse drug effects, myocardial infarction, toxic exposure, infections, surgery, and electrolyte disorders to be identified [3]. Figure 3 outlines this stepwise approach.

## Conclusions

Given the increasing prevalence of pacemaker implantation locally and worldwide both internists and cardiologists need to be cognizant of the need to adopt the stepwise approach suggested by the ESC, including the importance of taking a thorough history. Moreover, these patients should be appropriately investigated with ambulatory monitoring when necessary and if indicated early cardiology consultation for pacemaker implantation. Given the increasing need for permanent pacemaker implantation locally as well as the gap in implantation rates when compared to developed countries, policies need to be adopted at a national level to increase the number of cardiac electrophysiologists, the number of centers offering cardiac electrophysiology services, and the availability of pacemakers.
